# Circulating Lipocalin-2 and Retinol-Binding Protein 4 Are Associated with Intima-Media Thickness and Subclinical Atherosclerosis in Patients with Type 2 Diabetes

**DOI:** 10.1371/journal.pone.0066607

**Published:** 2013-06-17

**Authors:** Yang Xiao, Aimin Xu, Xiaoyan Hui, Pengcheng Zhou, Xing Li, Hui Zhong, Weili Tang, Gan Huang, Zhiguang Zhou

**Affiliations:** 1 Diabetes Center, the Second Xiangya Hospital, and Institute of Metabolism and Endocrinology, Key Laboratory of Diabetes Immunology, Ministry of Education, Central South University, Hunan Key Laboratory of Medical Epigenomics, Changsha, Hunan, China; 2 Department of Medicine, the University of Hong Kong, Hong Kong, China; 3 Department of Pharmacology and Pharmacy, the University of Hong Kong, Hong Kong, China; 4 Research Center of Heart, Brain, Hormone, and Healthy Aging, the University of Hong Kong, Hong Kong, China; The University of Hong Kong, Hong Kong

## Abstract

**Background:**

The lipocalin family proteins, including lipocalin-2 and retinol-binding protein 4 (RBP4), are adipokines closely associated with obesity-related metabolic disorders. In this study, we evaluated the association of serum lipocalin-2 and RBP4 with intima-media thickness (IMT) and subclinical atherosclerosis in type 2 diabetic patients.

**Methods and Results:**

Serum levels of lipocalin-2 and RBP4 were measured in 284 type 2 diabetic patients. Subclinical atherosclerosis was assessed by IMT at carotid, femoral and iliac arteries with ultrasound. Patients with subclinical atherosclerosis showed significantly higher circulating concentrations of lipocalin-2 and RBP4 when compared to those without [112.9 (86.4 to 202.1) µg/L versus 77.2(55.0–150.4) µg/L, 37.1(32.3–40.8) mg/L versus 23.2(20.1–29.2) mg/L, respectively; *P* = 0.002, *P*<0.001, respectively]. Moreover, positive correlations were observed between carotid IMT and lipocalin-2 (r = 0.170, *P* = 0.018) or RBP4 (r = 0.132, *P* = 0.040), femoral IMT and lipocalin-2 (r = 0.160, *P* = 0.027), as well as between iliac IMT and RBP4 (r = 0.241, *P*<0.001). Multiple logistic regression analysis further demonstrated that these two adipokines were independent risk factors for subclinical atherosclerosis in type 2 diabetes.

**Conclusion:**

Circulating levels of lipocalin-2 and RBP4 are positively correlated with carotid IMT and subclinical atherosclerosis in type 2 diabetes, which suggests a potential role of these two lipid-binding chaperones in the pathogenesis of vascular complications of diabetes.

## Introduction

Atherosclerotic disease is the primary cause of myocardial infarction, stroke, and peripheral vascular disease, which remains as the leading cause of death worldwide [1]. Identification of atherosclerosis at the subclinical stages would essentially facilitate earlier selection of more effective treatments which may lead to a better prognosis. Currently, indexes including the intima-media thickness (IMT), flow mediated vasodilatation (FMD) (markers of endothelial dysfunction), arterial stiffness, and ankle-brachial index (ABI) are most commonly used in routine clinical practice to evaluate subclinical atherosclerosis [2]. Nevertheless, more convenient and reliable markers, such as serum biomarkers, for subclinical atherosclerosis are yet to be identified.

Diabetes is one of the most important risk factors for atherosclerosis [3]. In fact, 70% of the morbidity associated with type 2 diabetes is related to atherosclerosis. In light of this, a better understanding on the missing link between type 2 diabetes and atherosclerosis would help to design more effective strategies for early intervention of this disease.

Type 2 diabetes is commonly accompanied by the abnormal production of adipokines [4]. Notably, a number of adipokines have been implicated in vascular and atherothrombotic complications. The circulating levels of several pro-inflammatory adipokines are elevated in pro-atherothrombotic and atherothrombotic states [5]. Lipocalin-2 (also known as 24p3, neutrophil gelatinase-associated lipocalin and siderocalin) [6] and retinol binding protein 4 (RPB4) [7] are novel adipokines that contribute to insulin resistance and type 2 diabetes in obese rodents. Both of them belong to the lipocalin family since they both share a common tertiary structure known as the ‘lipocalin fold’ which facilitates the binding of hydrophobic small molecules such as lipids [8]. Recent studies in human and animals have shown that both lipocalin-2 [9], [10], [11] and RBP4 [7], [12] are modulators of insulin signaling and their levels are elevated in subjects with obesity, insulin resistance, or type 2 diabetes. However, their relevance to cardiovascular diseases is less investigated. A marked elevation in lipocalin-2 expression was detected within atherosclerotic plaques of atherosclerotic animal models [6]. Aigner F. et al. demonstrated that lipocalin-2 regulates the inflammatory response during ischemia and reperfusion of the transplanted heart [13]. In the case of RBP4, two studies reported that circulating RBP4 levels remarkably correlated with the degree of carotid IMT [14], [15]. Plasma RBP4 concentrations were higher in those subjects with previous clinical arteriosclerosis than in event-free subjects [16], [17].

The above findings indicate a potential involvement of these two lipocalin proteins in cardiovascular diseases. However, the regulatory roles of circulating lipocalin-2 and RBP4 in type 2 diabetic patients with or without subclinical atherosclerosis have not been reported yet. In this study, we assessed serum lipocalin-2 and RBP4 concentrations in patients with newly diagnosed type 2 diabetes in relation to subclinical atherosclerosis, as measured by carotid, femoral and iliac arteries.

## Methods

### Ethics statement

This study was performed with the approval of the Institutional Review Board (IRB) of the Second Xiangya Hospital, Central South University. The written informed consent was received from every subject.

### Participants

Total 284 type 2 diabetic patients were enrolled in the Diabetes Center at the Second Xiangya Hospital, Central South University from the Chinese National Tenth Five Tackling Key Project which was a multicenter study to evaluate the diabetic vasculopathy in newly-diagnosed type 2 diabetes. Diabetes mellitus was diagnosed according to the American Diabetes Association criteria [18] with the following inclusion criteria: (1) disease duration less than 1 year; (2) age from 35 to 70 years old; (3) body mass index (BMI) from 19 to 35 kg/m^2^; (4) no ketosis within the first 6 months after diagnosis; (5) no renal dysfunction; (6) 4-week wash out if treated with insulin sensitizers; (7) no application of antihypertensive drugs or lipid-lowering drugs within recent 2 months.

Hypertension was diagnosed when systolic blood pressure ≥ 140 mm Hg and/or diastolic blood pressure ≥ 90 mm Hg or on antihypertensive medication. Dyslipidemia was defined as having one or more of the following criteria: (1) fasting triglycerides (TG) ≥ 1.7 mmol/L; (2) HDL-cholesterol (HDL-C) < 1.30 mmol/L in female and < 1.0 mmol/L in male; (3) LDL-cholesterol (LDL-C) ≥ 3.4 mmol/L; and/or (4) already on lipid-lowering drug according to United States Adult Treatment Panel III [19]. Smoking referred to both current and former smokers, whereas nonsmoker referred to those who never smoked.

### Clinical and biochemical assessments

A complete physical examination was performed on each patient. Height was measured to the nearest 0.5 cm and weight to the nearest 0.1 kg. Patients were measured in socks, stockings or bare feet and light street clothing. BMI (body mass index) was calculated as weight/height^2^ (kg/m^2^). Waist circumference was measured midway between the lower rib margin and iliac crest. Blood pressure measurements were taken on the right arm with the subject seated for at least 10 min. Systolic and diastolic phase blood pressures were recorded to the nearest 2 mmHg.

After overnight fasting, a venous blood specimen was collected in the morning (around 8:00 am) for analysis of various biochemical parameters as follows. FPG (fasting plasma glucose) and 2hPG (2 h postprandial plasma glucose) were measured by hexokinase method on a Hitachi 7170 analyzer (Boehringer Mannheim, Mannheim, Germany). Serum cholesterol and TG levels were determined enzymatically on the Hitachi 7170 analyzer (Boehringer Mannheim). Serum insulin was assessed by chemiluminescence on a Bayer 180SE Automated Chemiluminescence Systems (Bayer AG, Leverkusen, Germany). Insulin resistance was estimated with the homeostasis model assessment index (HOMA-IR), calculated as fasting glucose (in millimoles per liter) times fasting insulin (in milliinternational units per liter) divided by 22.5 [20]. Serum lipocalin-2 and RBP4 were measured using ELISA kits with monoclonal antibodies established in our laboratory (Antibody and Immunoassay Services, www.antibody.hku.hk) as previously reported [11], [21]. The intraassay and interassay coefficients of variation for lipocalin-2 were 3.8%–6.0% and 3.1%–5.2%, respectively. The intraassay and interassay coefficients of variation for RBP4 were 4.1%–6.8% and 3.8%–7.2%, respectively.

### Vascular Ultrasound Measurement

Examinations for subclinical atherosclerosis were performed. High-resolution B-mode ultrasound (128XP/10 system; Acuson, Mountain View, California, USA) was used to measure the intima-media thickness (IMT) of the target arteries (common carotid, femoral and common iliac arteries on the right side). Linear transducers with frequency of 10 to 12 MHz were used. An anterolateral approach was used to longitudinally capture the right common carotid, femoral and common iliac arteries. The best image was selected to show the far wall intimal–lumen interface as a continuous straight line. Three determinations of IMT were made at 1 cm proximal to the bulb and at the site of greatest thickness. The values at each site were averaged, and the averaged IMT was used as the representative value for each individual. Plaque was defined as IMT ≥1.3 mm or a focal protrusion into the lumen with a thickness of at least 50% more than adjacent intima-media complex. Subclinical atherosclerosis was defined as IMT>1.0 mm and/or with plaque on one or more of the three arteries (common carotid artery, femoral artery and common iliac artery) but without clinical manifestations [22], [23].

### Statistical analysis

All statistical analyses were performed with Statistical Package for Social Science Version 16.0 (SPSS 16.0, Inc., Chicago, IL). Data were expressed as mean ± SD or median with interquartile range as appropriate. Data that were not normally distributed, as determined using Kolmogorov-Smirnov test, were naturally logarithmically transformed before analysis. Correlations between lipocalin-2 or RBP4 and biochemical variables were analyzed with Pearson correlation. Comparisons between groups were performed using χ^2^ tests for categorical variables and independent-samples T test or univariate general linear model for continuous variables as indicated. Multiple logistic regression analysis was done to determine independent factors of subclinical atherosclerosis, and included variables which were significantly different between patients with and without subclinical atherosclerosis and were biologically likely to be related with atherosclerotic status. Two-sided *P* values <0.05 were considered significant.

## Results

The anthropometric and biochemical characteristics of the type 2 diabetic patients are summarized in Table 1. Compared with patients without subclinical atherosclerosis, patients with it had significantly higher carotid IMT, femoral IMT, iliac IMT, systolic blood pressure and elder age. Notably, serum lipocalin-2 and RBP4 levels were significantly higher in patients with subclinical atherosclerosis when compared to those in patients without [112.9 (86.4 to 202.1) µg/L versus 77.2(55.0–150.4) µg/L, 37.1(32.3–40.8) mg/L versus 23.2(20.1–29.2) mg/L, respectively; *P* = 0.002, *P*<0.001, respectively].

**Table 1:** Anthropometric and biochemical characteristics of type 2 diabetic subjects subdivided into subAS and non-subAS subjects.

Parameters	subAS(n = 78)	Non-subAS(n = 206)	*P*
Age (yr)	56.9±8.3	53.4±8.4	0.002
Sex (M/F)	38/40	106/100	NS
Smoking (%, Never-smoker/Former and current smoker)	61.2/38.8	62.6/37.4	NS
BMI (kg/m^2^)	25.1±2.8	24.5±2.8	NS
Waist circumference (cm)			
Male	89.4±9.0	89.3±8.0	NS
Female	84.2±7.9	83.6±7.2	
Systolic blood pressure (mmHg)	123±17.6	118±17.5	0.037
Diastolic blood pressure (mmHg)	75±11.1	76±10.3	NS
FPG (mmol/liter)	7.9±2.8	7.5±2.3	NS
2hPG (mmol/liter)	12.7±4.8	11.7±4.3	NS
TG (mmol/liter) †	1.6(1.1–2.3)	1.6(1.2–2.6)	NS
LDL-C (mmol/liter)	3.2±1.1	3.0±0.9	NS
HDL-C (mmol/liter)	1.3±0.4	1.3±0.4	NS
Fasting insulin (mIU/liter) †	12.4 (8.2–18.3)	14.0 (9.0–19.0)	NS
Carotid IMT (mm)	0.94±0.34	0.70±0.11	<0.001
Femoral IMT (mm)	0.97±0.33	0.70±0.11	<0.001
Iliac IMT (mm)	1.13±0.28	0.76±0.10	<0.001
Lipocalin-2 (µg/liter) †	112.9(86.4–202.1)	77.2(55.0–150.4)	0.002
RBP4 (mg/liter) †	37.1(32.3–40.8)	23.2(20.1–29.2)	<0.001
Presence of hypertension (%)	26.9	22.3	NS
Presence of dyslipidemia (%)	72.7	69.9	NS

† Log transformed before analysis.

subAS, subclinical atherosclerosis; M, male; F, female; BMI, body mass index; FPG, fasting plasma glucose; 2hPG, 2 h postprandial glucose; TG, triglycerides; HDL-C, high-density lipoprotein cholesterol; LDL-C, low-density lipoprotein cholesterol; IMT, intima-media thickness; RBP4, retinol-binding protein 4.

**Figure 1:**
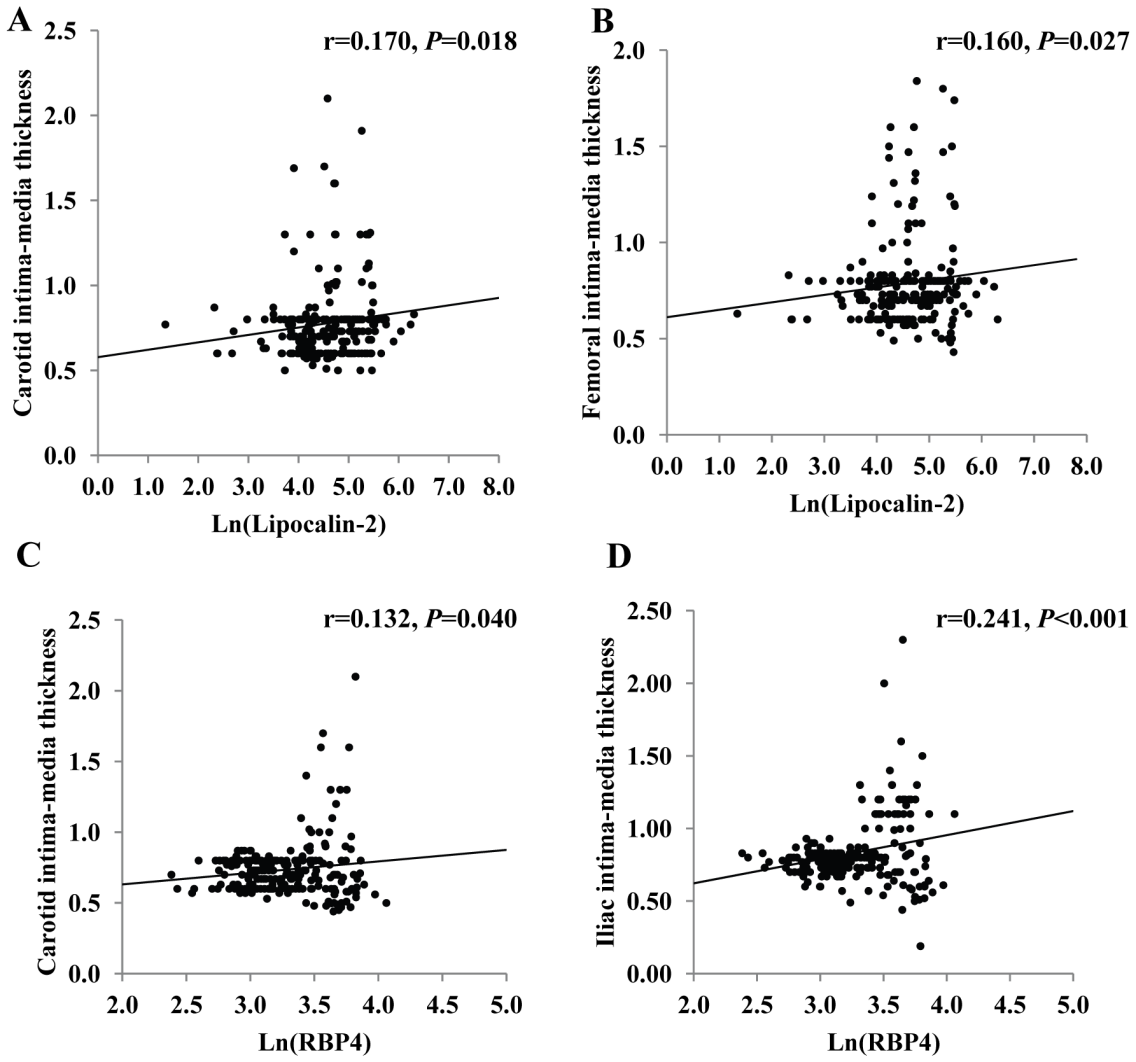
Correlations between lipocalins and IMTs. Correlations between serum Lipocalin-2 and (A) carotid IMT (n = 225) or (B) femoral IMT (n = 223) in type 2 diabetic patients; correlations between serum RBP4 and (C) carotid IMT (n = 244) or (D) iliac IMT (n = 242) in type 2 diabetic patients. Ln, natural logarithm.

Serum lipocalin-2 levels correlated positively with BMI, waist circumference, TG and fasting insulin. Serum RBP4 levels correlated positively with systolic blood pressure (Table 2). In the whole study group, positive correlations were observed between carotid IMT and lipocalin-2 (r = 0.170, *P* = 0.018) or RBP4 (r = 0.132, *P* = 0.040), femoral IMT and lipocalin-2 (r = 0.160, *P* = 0.027), as well as between iliac IMT and RBP4 (r = 0.241, *P*<0.001, Figure 1). In addition, serum lipocalin-2 correlated closely with RBP4 (r = 0.231, *P* = 0.003).

**Table 2:** Correlation of serum lipocalin-2 and retinol-binding protein 4 levels with other parameters.

Parameters	Lipocalin-2†		RBP4†	
	r	*P*	r	*P*
Lipocalin-2†			0.231	0.003
RBP4†	0.231	0.003		
Age	0.013	NS	0.015	NS
Sex	–0.126	NS	–0.051	NS
BMI	0.190	0.008	0.072	NS
Waist circumference	0.171	0.018	–0.024	NS
Systolic blood pressure	–0.030	NS	0.149	0.020
Diastolic blood pressure	0.128	NS	0.085	NS
FPG	–0.065	NS	0.012	NS
2hPG	0.034	NS	0.030	NS
TG†	0.170	0.019	0.056	NS
HDL-C	–0.103	NS	–0.078	NS
LDL-C	–0.087	NS	0.024	NS
Fasting insulin†	0.165	0.022	–0.051	NS
HOMA-IR†	0.118	NS	–0.044	NS

† Log transformed before analysis.

r indicates Pearson correlation coefficient; RBP4, retinol-binding protein 4; BMI, body mass index; FPG, fasting plasma glucose; 2hPG, 2 h postprandial glucose; TG, triglycerides; HDL-C, high-density lipoprotein cholesterol; LDL-C, low-density lipoprotein cholesterol.

Using a multiple logistic regression model, independent significant risk factors for subclinical atherosclerosis were identified, including age, sex, BMI, smoking status, FPG, fasting insulin, presence of hypertension, presence of dyslipidemia and lipocalin-2 or RBP4 (Table 3). Serum lipocalin-2 was independently associated with subclinical atherosclerosis in type 2 diabetes (OR 2.10, 95% confidence interval (CI) 1.06–4.16, *P* = 0.033), together with age (*P*<0.001), BMI (*P* = 0.001) and FPG (*P* = 0.016). Similarly, if lipocalin-2 was replaced by RBP4 in the model, RBP4 was independently associated with subclinical atherosclerosis in type 2 diabetes (OR 1.16, 95% CI 1.10–1.22, *P*<0.001). If FPG was replaced by 2hPG in both models, lipocalin-2 or RBP4 remained as a significant factor associated with subclinical atherosclerosis in type 2 diabetes (OR 2.28, 95% CI 1.08–4.83, *P* = 0.031; OR 1.15, 95% CI 1.09–1.21, *P*<0.001, respectively). When waist circumference replaced BMI in all models, the results were similar (OR 2.29, 95% CI 1.17–4.49, *P* = 0.016; OR 1.16, 95% CI 1.10–1.23, *P*<0.001, respectively). The significant association between lipocalin-2 or RBP4 and subclinical atherosclerosis remained unchanged after the HOMA-IR was taken into consideration when performing regression (OR 2.18, 95% CI 1.08–4.38, *P* = 0.029; OR 1.17, 95% CI 1.11–1.24, *P*<0.001, respectively).

**Table 3:** Multiple logistic regression analysis showing the parameters with significant independent associations with subclinical atherosclerosis in type 2 diabetes.

	Model 1		Model 2	
Parameters	OR(95%CI)	*P*	OR(95%CI)	*P*
Age	1.16(1.08–1.24)	<0.001	1.04(0.98–1.11)	0.147
Sex (female)	2.90(0.82–10.22)	0.097	2.66(0.55–12.79)	0.223
BMI	1.44(1.17–1.77)	0.001	1.07(0.88–1.29)	0.513
Smoking	1.58(0.41–6.17)	0.508	1.48(0.28–7.75)	0.643
FPG	1.25(1.04–1.49)	0.016	1.09(0.90–1.31)	0.376
Fasting insulin †	0.44(0.18–1.10)	0.080	0.55(0.23–1.31)	0.180
Presence of hypertension	0.50(0.16–1.53)	0.223	1.06(0.38–2.92)	0.917
Presence of dyslipidemia	1.09(0.38–3.08)	0.878	1.15(0.43–3.08)	0.778
Lipocalin-2 †	2.10(1.06–4.16)	0.033	–	–
RBP4	–	–	1.16(1.10–1.22)	<0.001

† Log transformed before analysis.

Models included age, sex, BMI, smoking status, fasting insulin, FPG, presence of hypertension, presence of dyslipidemia and lipocalin-2 (model 1) or RBP4 (model 2).

## Discussion

Atherosclerosis is a systemic disease affecting multiple territories in the arterial wall. Subclinical atherosclerosis, as classified by 3 vessel beds involved (including carotid, femoral and iliac arteries), is used to diagnose generalized atherosclerosis and considered as a better predictor for cardiovascular events [22], [23]. Our study revealed an independent correlation between subclinical atherosclerosis and serum lipocalin-2 and RBP4 levels in type 2 diabetic patients, suggesting that these lipocalins might be involved in the early stage of diabetic vascular complications. Serum lipocalin-2 and RBP4 levels were significantly increased in patients suffering from subclinical atherosclerosis. Serum lipocalin-2 levels correlated positively with individual components of subclinical atherosclerosis carotid IMT and femoral IMT, while serum RBP4 levels positively correlated with carotid IMT and iliac IMT. Multiple logistic regression analysis revealed that both serum lipocalin-2 and RBP4 were independent risk factors for subclinical atherosclerosis in type 2 diabetes, and the impact of lipocalin-2 or RBP4 on atherosclerosis was independent of age, sex, BMI, and other classical cardiovascular risk factors.

The mechanisms by which these two lipocalin proteins mediate the progression from type 2 diabetes to atherosclerosis are unknown. We propose that they might serve as a relay between inflammation and lipid metabolism in the transition from type 2 diabetes to atherosclerosis. It is now generally believed that diabetes evokes inflammation, vasoconstriction and thrombosis that collectively contribute to atherosclerosis [3]. In particular, recent advances in basic science have established a fundamental role of inflammation in almost every stage of atherosclerosis from endothelial dysfunction to initiation, progression, and ultimately, destabilization and rupture of plaques [24].

The pro-inflammatory features of lipocalin-2 and RBP4 have been highlighted by several studies. Expression of lipocalin-2 is induced in both acute and chronic inflammation [25]. The lipocalin-2 promoter contains binding sites for a crucial pro-inflammatory transcription factor, nuclear factor κB (NF-κB), and lipocalin-2 was found to be highly induced in the intima following angioplasty, as a consequence of NF-κb activation in a rat carotid artery injury model [26], [27]. More importantly, there are evidences suggesting that lipocalin-2 may participate in the development of atherosclerosis by inducing inflammation. Lipocalin-2 plays critical roles in regulating the expression of tumor necrosis factor-α (TNF-α) in fat tissues, partly through upregulating 12-lipoxygenase expression and activity [28], [29]. In both atherosclerotic plaques and the intima of injured vessels, lipocalin-2 colocalizes with matrix metalloproteinase 9 (MMP-9), a key protease in inflammation and atherosclerosis [30]. Associations between RBP4 and markers of inflammation have also been implicated by a few clinical studies. Serum RBP4 levels were increased and correlated with subclinical inflammation in childhood obesity [31], while in adipose tissue, mRNA level of RBP4 was positively linked to inflammatory markers rather than insulin resistance [32]. Furthermore, high plasma RBP4 has been associated with systemic inflammation in chronic kidney disease in the absence of obesity and diabetes [33]. Taken together, it is possible that lipocalin-2 and RBP4 mediate atherogenesis via enhancing vascular inflammation in type 2 diabetes.

Hyperlipidemia is another risk factor for atherosclerosis. Notably, lipocalins possess lipid-binding properties [34], [35], [36], which may possibly mediate the hyperlipidemia evoked-inflammation and atherosclerosis by acting as lipid sensors. It is intriguing to investigate in the future whether the lipid binding capacity of these lipocalins is mandatory for their pro-inflammatory and pro-atherogenesis activities. In light of this, lipocalin-2 and RBP4 might serve as critical links between lipid metabolism and inflammation in atherogenesis.

Consistent with previous findings [9], [11], we found that circulating lipocalin-2 levels are related to BMI, waist circumference, TG and fasting insulin. Nevertheless, circulating RBP4 levels were exclusively related to systolic blood pressure. The relationship between RBP4 and hypertension might be an underlying cause in reinforcing the risk of atherogenesis [14]. We also observed a close relationship between lipocalin-2 and RBP4. However, their distinct correlations with various metabolic parameters, suggest that lipocalin-2 and RBP4 should have different patterns of regulation and there exists a complex interconnection between these two lipocalin proteins and metabolic disorders.

Our study has several limitations. Firstly, due to the relatively small sample size and the cross-sectional design, the predictive value of lipocalin-2 or RBP4 needs to be confirmed in a larger cohort. Secondly, ultrasound can assess only the structural changes in arteries, but not the vascular inflammatory status or qualitative assessment. Therefore, fluorodeoxyglucose (FDG) positron emission tomography (PET) and virtual histology-IVUS may provide more precise data on the vascular inflammation and plaque components.

In conclusion, our study suggests that serum lipocalin-2 or RBP4 levels reflect subclinical atherosclerosis in adults with newly diagnosed type 2 diabetes. These findings support the roles of these two lipid-binding chaperones in the pathogenesis of vascular complications of diabetes, and provide possible perspective for using lipocalin-2 and RBP4 as biomarkers for early detection of high-risk individuals developing cardiovascular diseases and potential therapeutic targets for atherosclerosis.
